# The influence of increasing color variety on numerosity estimation and counting

**DOI:** 10.3758/s13423-024-02625-x

**Published:** 2025-01-03

**Authors:** Qi Li, Guo Ting, Yuichiro Kikuno, Yokosawa Kazuhiko

**Affiliations:** 1 Institute for the Future of Human Society, Kyoto University, 46 Shimoadachi-Cho, Yoshida Sakyo, Kyoto, 606-8501 Japan; 2 Research Center for Language and Cognition, Ningbo University of Technology, Ningbo, China; 3 Department of Psychology, Kyoto Notre Dame University, Kyoto, Japan; 4 Department of Business and Information Science, Japan International University, Tsukuba, Japan

**Keywords:** Numerical cognition, Attention, Estimation, Counting, Color, Spatial arrangement

## Abstract

Previous research has suggested that numerosity estimation and counting are closely related to distributed and focused attention, respectively (Chong & Evans, *WIREs Cognitive Science*, *2*(6), 634–638, 2011). Given the critical role of color in guiding attention, this study investigated its effects on numerosity processing by manipulating both color variety (single color, medium variety, high variety) and spatial arrangement (clustered, random). Results from the estimation task revealed that high color variety led to a perceptual bias towards larger quantities, regardless of whether colors were clustered or randomly arranged. This implies that distributed attention may engage in a global assessment of color richness, with less emphasis on spatial arrangement. In contrast, the effect of color on counting was influenced by spatial arrangement: performance improved with clustered colors but declined with random color distribution. This indicates that color interacts with spatial information to modulate focused attention during serial numerosity processing. Taken together, our findings provide new insights into the interaction between numerical cognition and attention, highlighting the need for theories and models of numerical cognition to take into account feature variety and contextual factors, such as the spatial arrangement of features. Additionally, in light of the widespread diversity in real-world environments, our findings could inform strategies to enhance behavioral adaptation to varying environmental conditions.

**Supplementary Information:**

The online version contains supplementary material available at 10.3758/s13423-024-02625-x.

## Introduction

Quantity information is a fundamental aspect of our environment. The ability to perceive and manipulate numerical quantities supports our action in a wide range of daily tasks. Small quantities, typically four items or fewer, can be perceived rapidly and accurately (Kaufman et al., 1949). For large quantities, we either estimate the approximate number at a glance or counting each item to determine the exact number.

Numerical cognition and attention are closely linked. Chong and Evans (2011) proposed that distributed attention enables estimation, while focused attention supports counting. This aligns with Treisman’s view that attention shapes the visual system to accommodate various perceptual tasks using distributed and focused modes for global and local processing, respectively (Treisman, 2006). These attention modes provide a framework for interpreting experimental findings in numerical cognition. For example, Treisman (2006) showed that participants could easily estimate numerical proportions based on individual features but struggled with feature combinations, highlighting different approaches to numerical coding by varying attention modes. Supporting this framework, Demeyere and Humphreys (2007) described a patient with simultanagnosia who had severe counting difficulties but relatively intact magnitude estimation abilities, indicating a dissociation between focused and distributed attention functions.

Within the framework of two modes of attention, it is plausible that factors affecting distributed and focused attention impact numerosity estimation and counting. Empirical studies in visual search demonstrate that specific basic features (e.g., color, orientation, size) effectively influence the deployment of visual attention and search performance (Duncan & Humphreys, 1989; Treisman & Gormican, 1988). Some theories, such as the Guided Search Theory (Wolfe, 2007), suggest that variations in the quality of guidance by features lead to different search performance patterns, with color recognized as a key attention-guiding feature (Brawn & Snowden, 1999; Treisman & Souther, 1985).

However, limited data exist on how color affects numerical cognition. Halberda et al. (2006) reported that participants could estimate subset quantities based on shared color, with total quantity estimation unaffected by color variety. Cordes et al. (2014) also found that participants could focus on a specific color subset to estimate its quantity, but they perceived a greater total number of items in two-color displays compared to single-color displays. While these studies suggest that subset estimation based on color is feasible and highlight the relationship between attentional selection and numerical perception, findings on superset estimation are inconsistent, with one study showing no effect and another indicating a color-induced bias, leaving the influence of color on superset numerosity estimation unclear. This study systematically investigated the effects of color on numerosity estimation by manipulating color variety and the spatial arrangement of colors. We further extended our investigation to include a counting task, aiming for a more comprehensive examination of how basic features influence different types of numerical processing.

## Experiment 1

In this experiment, we explored how object color affects numerosity estimation by varying color variety from single color to medium variety (four colors) and high variety (eight colors). Previous research suggests that people often associate larger quantity with greater variety (Broniarczyk et al., 1998). The opposite might also be true: greater variety could be seen as indicating a larger quantity, making sets with various colors appear more numerous than those with a single color. We also examined whether the spatial arrangement of colors (clustered vs. random) influences numerosity estimation, as it might affect color variety perception. However, some researchers argue that numerosity encoding generates an abstract representation unaffected by non-numerical attributes (Dehaene & Changeux, 1993), suggesting that increased color variety may not influence numerosity estimation.

### Method

#### Participants

We prespecified a sample size of 30 participants, which aligns with the upper range used in previous studies on numerosity estimation (Cordes et al., 2014; Halberda et al., 2006). To confirm the adequacy of our sample size, we also conducted a power analysis using G*Power 3.1 (Faul et al., 2007). This analysis indicated that a minimum sample size of *n* = 15 would be required to achieve 80% power for detecting a moderate effect size *f* of 0.25, with an alpha level of 0.05, in a 2 × 3 × 5 within-participants repeated-measures analysis of variance (ANOVA).

Thirty undergraduate students (21 females, nine males) aged 18–22 years (*M* = 19.47, *SD* = 1.04) from Ningbo University of Technology participated in the experiment for monetary compensation. All participants had normal color vision and either normal or corrected-to-normal visual acuity, and provided written informed consent before participating. The experiment protocol received approval from the Institutional Review Board of Ningbo University of Technology and all procedures were conducted in accordance with relevant guidelines and regulations.

#### Apparatus

The experiment was conducted in a darkened testing room. Participants were seated 75 cm away from a 24-in. LCD monitor (ASUS VG248; 144-Hz refresh rate; 1,920 × 1,080). To maintain a stable viewing distance, chinrest, forehead rest, and temple stabilizers were used. Visual stimuli were presented using the Psychophysics Toolbox (Brainard, 1997; Pelli, 1997) implemented in Matlab.

#### Stimuli and design

**Fig. 1:**
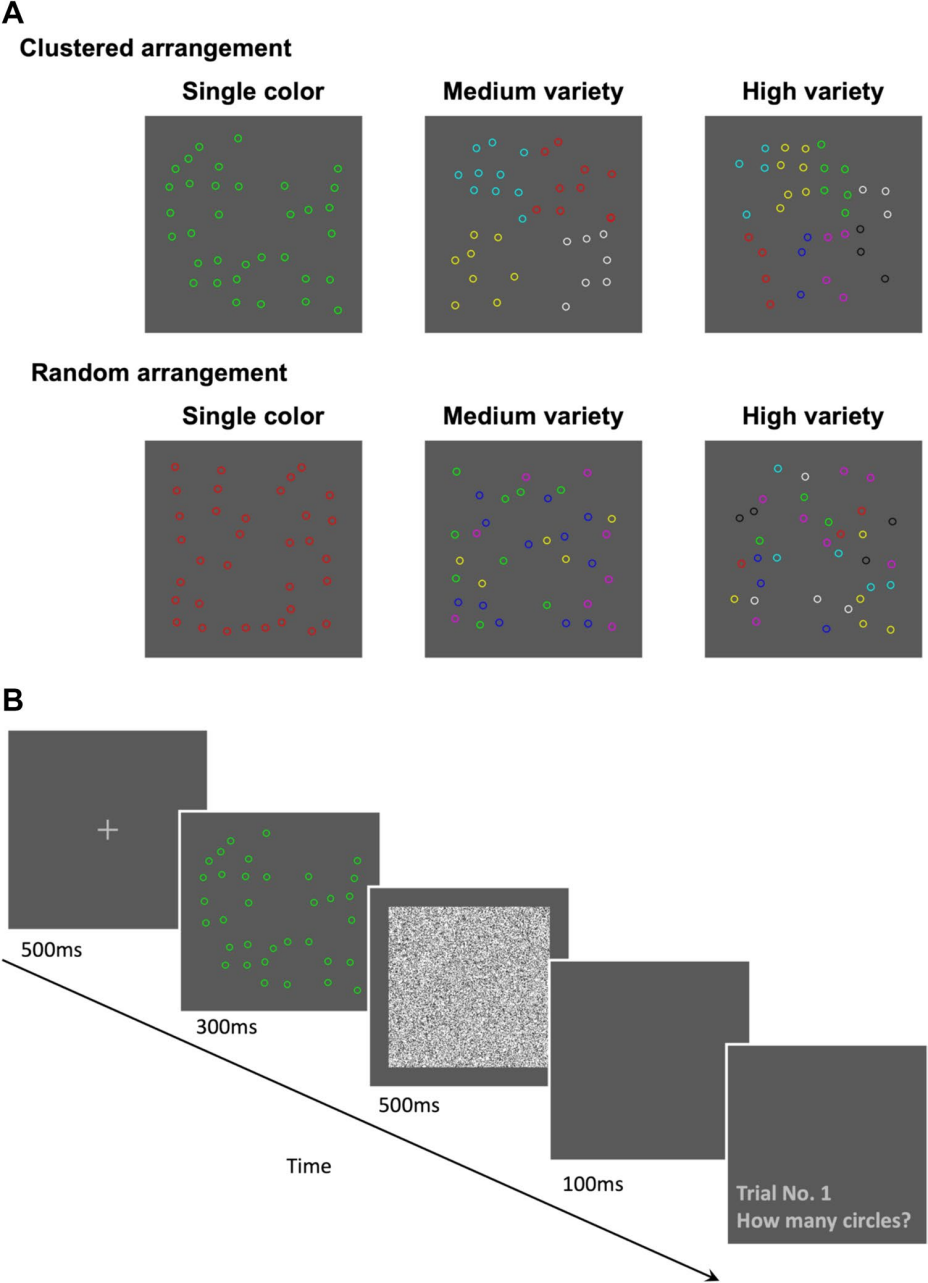
Stimuli and task in Experiment 1. (**A**) Sample stimulus arrays of single color, medium variety (four colors), and high variety (eight colors) in both clustered and random arrangements. (**B**) The sequence of trial events in the estimation task

As illustrated in Fig. 1A, the estimation displays comprised colored circles (diameter = 0.6°) presented within a 12.5° × 12.5° region against a dark gray background (RGB: 64, 64, 64). The colored circles were randomly positioned on an invisible 8 × 8 grid, with each circle subject to random jittering within a range of 0.8°. Color selection was based on the work of Halberda et al. (2006), which included red, green, blue, yellow, magenta, and cyan. To broaden the color variety, we also included black and white. For the specific RGB settings, we referenced Demeyere and Humphreys (2007) and utilized maximum and minimum RGB values as follows: red (RGB: 255, 0, 0), green (RGB: 0, 255, 0), blue (RGB: 0, 0, 255), yellow (RGB: 255, 255, 0), magenta (RGB: 255, 0, 255), cyan (RGB: 0, 255, 255), black (RGB: 0, 0, 0), and white (RGB: 255, 255, 255). Colors were randomly selected on each trial to minimize any effect of differential color saliency.

We manipulated three factors in the experiment: color variety, spatial arrangement, and numerosity. For color variety, participants experienced three conditions: single color, medium variety (four colors), and high variety (eight colors), with the single-color condition serving as a baseline measure for performance. In the medium- and high-variety conditions, we applied the following constraints on the quantity of each color: (1) the minimum quantity of circles in each color was the nearest integer greater than or equal to N/2C, while the maximum quantity was 64/C, where N is the total number of circles and C is the number of colors (4 or 8); (2) the quantities of circles in each color were not entirely equal, ensuring a disparity in the quantities of at least two colors. These constraints facilitated substantial variations in the quantities of each color while avoiding extremes and preventing entirely equal amounts. For spatial arrangement, there were two types: clustered and random (Fig. 1A). In the clustered arrangement, circles of the same color were grouped together, while in the random arrangement, circles of different colors were scattered randomly across the display. For numerosity, five target values (13, 20, 27, 34, and 41 circles) and 16 filler values (12, 14, 17, 19, 21, 23, 26, 28, 30, 33, 35, 38, 40, 42, 45, and 47 circles) were used to maintain unpredictability in quantity.

#### Procedure

As shown in Fig. 1B, each trial began with a light gray central fixation (“ + ”, 0.6° × 0.6°, RGB: 125, 125, 125) presented for 500 ms, followed by an estimation display showing an array of circles for 300 ms. Subsequently, a mask display featuring random white dots covering the 12.5° × 12.5° area where the circles were presented appeared and lasted for 500 ms. After a 100-ms blank, a response display appeared, asking participants to report their estimate. Participants were instructed to estimate the numbers of circles as accurately as possible and orally report their estimate upon the appearance of the response display. Following their oral response, participants pressed a key to proceed to the next trial.

Participants completed the clustered and random arrangement conditions on separate days. Half of the participants performed the clustered session on day one and the random session on day two, while the other half followed the reverse order. The experiment consisted of a total of 576 trials (288 clustered trials and 288 random trials). Within both the clustered and random arrangements, there were 96 trials for each of the single-color, medium-variety, and high-variety conditions. Within each color variety condition, there were 16 trials for any of the five target numerosities, and one trial for any of the 16 filler numerosities. All trial types occurred in a random order throughout both clustered and random sessions. Each experimental session included 15 practice trials (five trials for each of the three color variety conditions) before the actual experiment. The numerosities used in the practice trials (15, 22, 29, 36, and 43) differed from those used in the actual experiment. Accurate numerosity feedback was provided after each response during the practice phase, whereas no feedback was given during the actual experiment.

#### Data analysis

Trials where participants proceeded to the next trial without responding due to accidental key-presses were excluded from the analyses. Estimation accuracy was evaluated using a score derived from the difference between the estimated value and the exact value. Positive scores indicate overestimation, while negative scores indicate underestimation. Estimation scores were analyzed using a three-way repeated-measures ANOVA with spatial arrangement (clustered, random), color variety (single color, medium variety, high variety), and numerosity (13, 20, 27, 34, 41) as within-participant factors. Shaffer’s modified sequentially rejective Bonferroni correction was applied to adjust *p*-values where appropriate.

While the estimation score serves as the primary indicator of participants' accuracy and bias tendencies, we also calculated the standard deviations of estimation errors for each participant and analyzed these data with the same three-way ANOVA used for the estimation scores to evaluate the consistency and precision of their estimations across different conditions. Since this ANOVA revealed no significant effects of color variety or interactions with other factors, we report the results of the standard deviation of estimation errors in the Appendix in the Online Supplementary Material for conciseness.

### Results

**Fig. 2:**
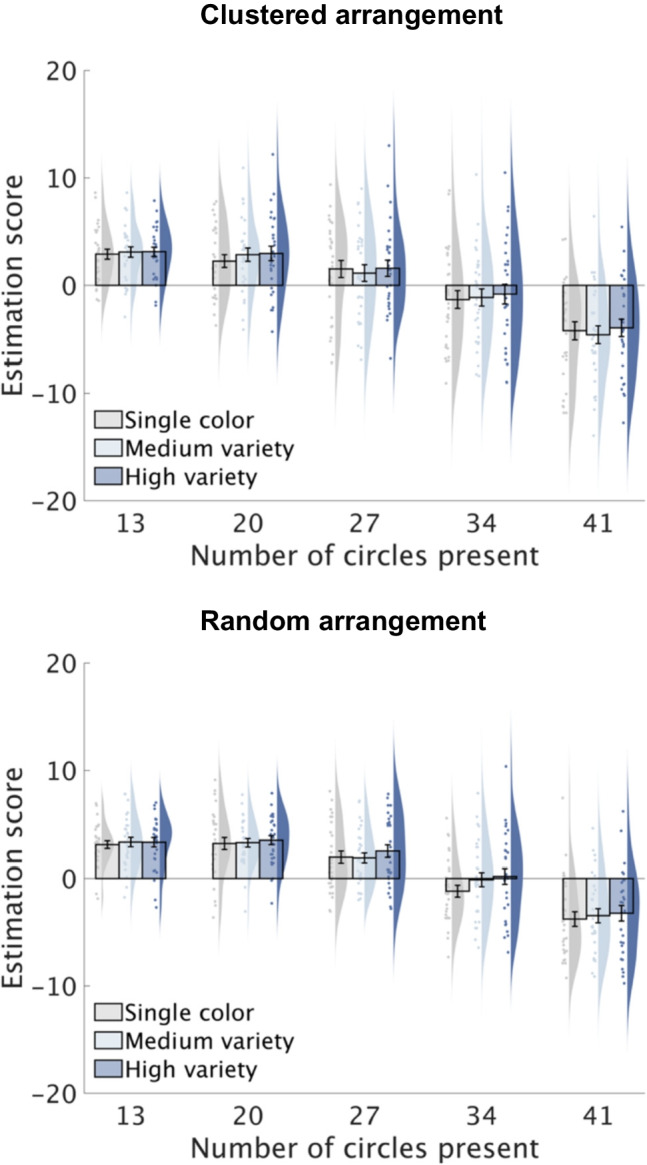
Estimation scores (estimated value – exact value) in Experiment 1. Bar plots depict the group means for each condition with error bars representing standard errors. Individual means are shown as scatter points, and their distribution is represented using Kernel density estimation

The estimation scores (estimated value – exact value) are shown in Fig. 2. A 2 × 3 × 5 ANOVA yielded a significant main effect of color variety (*F*(2, 58) = 4.45, *p* = 0.016, ${\upeta }_{\text{p}}^{2}$ = 0.13). Post hoc contrasts revealed that high-variety trials had higher scores than single-color trials (*t*(29) = 2.65, *p* = 0.038), indicating that high-variety displays appeared more numerous, with estimates increasing by 0.77–4.12%. High-variety trials also showed a marginal trend towards higher scores than medium-variety trials (*t*(29) = 1.94, *p* = 0.063), while the difference between medium-variety and single-color trials did not approach significance (*p* > 0.23). There was also a main effect of numerosity (*F*(4, 116) = 137.29, *p* < 0.001, ${\upeta }_{\text{p}}^{2}$ = 0.83), with scores decreasing as numerosity increased (*p*s < 0.001), except between numerosities 13 and 20 (*p* > 0.55). No significant main effect of spatial arrangement or interactions among factors were observed, indicating that the effect of color variety was comparable for clustered and random arrangements.

### Discussion

The observed increase in underestimation with larger numerosities aligns with earlier research indicating that larger quantities are often more underestimated (Krueger, 1972, 1982). The tendency to overestimate small-numerosity arrays while underestimating larger ones is consistent with previous findings (Li et al., 2018; Xiang et al., 2021) and may stem from the central tendency effect, where perceptual judgments bias toward the center of the stimulus distribution (Hollingworth, 1910; Stevens & Greenbaum, 1966).

A key finding was that high-variety displays consistently appeared more numerous than single-color displays, regardless of spatial arrangement. This suggests that estimating numerosity is influenced by color variety during brief presentations, with distributed attention enabling holistic diversity evaluation and diminishing the influence of spatial arrangement. Although increasing color variability might disrupt distributed attention, given that color serves as a strong selection cue (Zhang & Luck, 2009), our results did not show evidence supporting this notion. Estimation accuracy did not uniformly worsen in the high-variety condition. The observed numerical bias affected accuracy differently depending on numerosity. For small numerosities, this bias decreased accuracy due to exaggerated overestimation, whereas for large numerosities, it improved accuracy by mitigating underestimation. Additionally, our analysis of estimation precision using the standard deviations of estimation errors revealed no effect of color variety (see Appendix in the Online Supplementary Material), indicating that precision did not systematically decrease with greater variety. It seems that distributed attention remains effective in multi-color displays, with participants rapidly registering color diversity and using it as a heuristic for inferring a larger number of objects. Notably, attention can be allocated through various mechanisms, including spatial (Posner et al., 1980), feature-based (Treue & Martinez-Trujillo, 1999), and object-based selection (Duncan, 1984). In our experiment, the consistent stimulus presentation within a fixed area likely encouraged spatial selection of the entire area, enabling effective object selection despite color variety.

## Experiment 2

In Experiment 2, we investigated how color affects serial counting. Previous research suggests that spatial segmentation of stimulus displays can guide focused attention and facilitate counting (Li et al., 2018). Here, color clusters naturally segmented the displays. If color clusters aid the allocation of focused attention, counting performance should improve. Randomly arranged colors might also enhance counting by aiding object individuation. However, increased color variation could introduce redundancy and noise, potentially impairing counting.

### Method

Unless stated otherwise, the method in Experiment 2 was identical to that of Experiment 1.

#### Participants

A group of 30 new participants (five females, 25 males), aged 18–23 years (*M* = 20.87, *SD* = 1.66), participated in Experiment 2.

#### Procedure

**Fig. 3:**
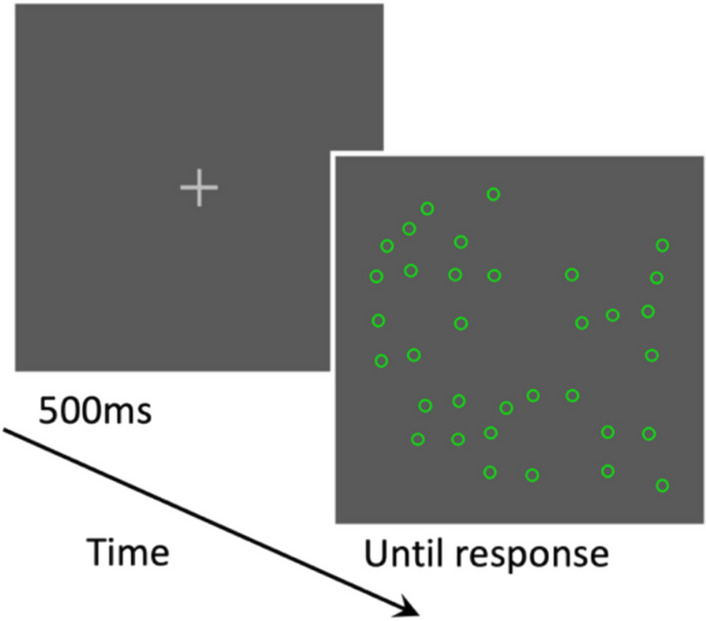
The sequence of trial events in the counting task

Figure 3 illustrates the sequence of trial events in the counting task. Trials began with a light gray central fixation cross lasting 500 ms. Following the fixation display, a counting display consisting of an array of circles appeared. This display remained present until the response key (the “5” key on the numeric keypad) was pressed, after which it was replaced by a response display instructing participants to report the number of circles orally. Participants were instructed to count the circles as quickly and accurately as possible and to press the response key as soon as they finished counting.

#### Data analysis

Response times (RTs) that fell outside three standard deviations from the individual mean for each combination of conditions were treated as outliers and removed. The RT analysis included only correct trials and employed a three-way ANOVA with spatial arrangement (clustered, random), color variety (single color, medium variety, high variety), and numerosity (13, 20, 27, 34, 41) as within-subject factors. The error rate, defined as the proportion of incorrectly counted trials, underwent a similar 2 × 3 × 5 ANOVA as that used for the RT data.

### Results

**Fig. 4:**
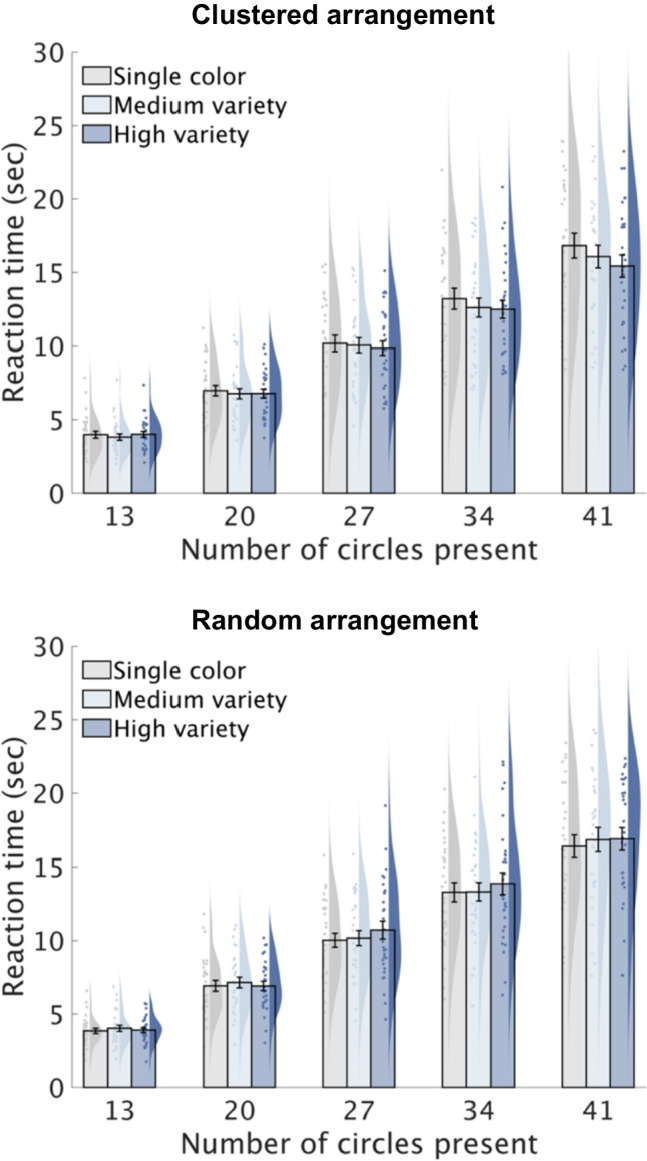
Reaction time in Experiment 2. Bar plots depict the group means for each condition with error bars representing standard errors. Individual means are shown as scatter points, and their distribution is represented using Kernel density estimation

**Fig. 5:**
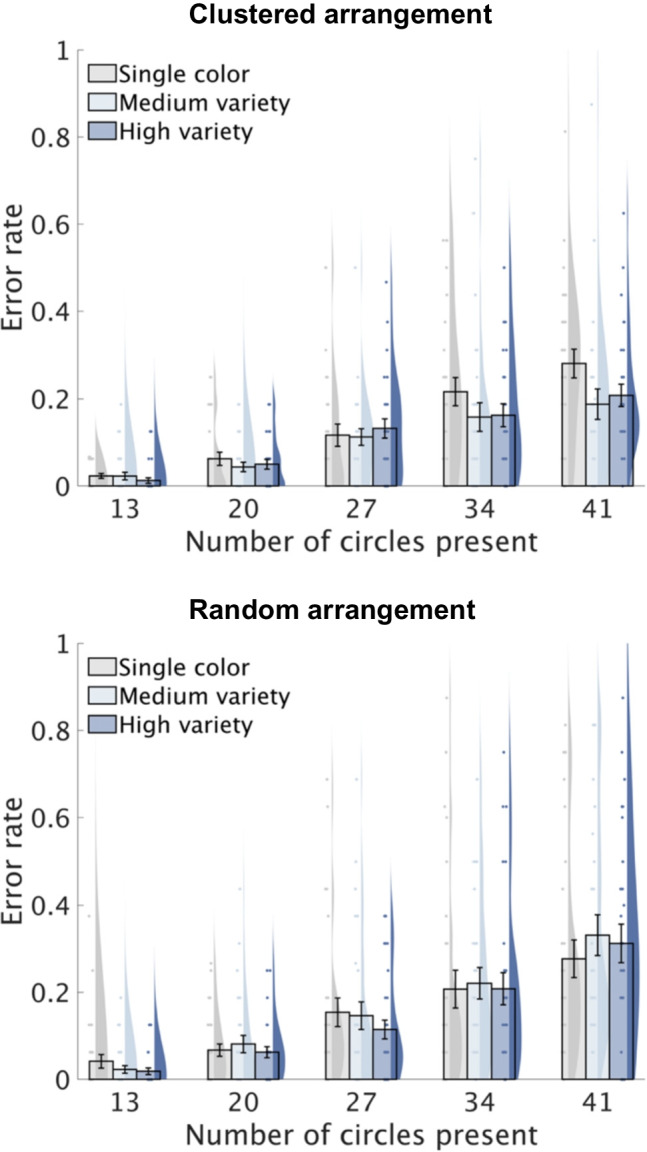
Error rates in Experiment 2. Bar plots depict the group means for each condition with error bars representing standard errors. Individual means are shown as scatter points, and their distribution is represented using Kernel density estimation

Table 1 presents the ANOVA results for RTs and error rates, with Figs. 4 and 5 showing descriptive data. Below, we summarize the key findings regarding the three-way interaction.

**Table 1:** Three-way repeated-measures ANOVA results for response times (RTs) and error rates

Variable	*df1*	*df2*	RT			Error rate		
			*F*	*p*	${\upeta }_{\text{p}}^{2}$	*F*	*p*	${\upeta }_{\text{p}}^{2}$
Spatial arrangement	1	29	1.14	0.294	0.04	2.42	0.130	0.08
Color variety	2	58	0.48	0.620	0.02	2.77	0.071 +	0.09
Numerosity	4	116	408.20	< 0.001***	0.93	54.28	< 0.001***	0.65
Spatial arrangement × Color variety	2	58	11.83	< 0.001***	0.29	7.88	< 0.001***	0.21
Spatial arrangement × Numerosity	4	116	2.42	0.053 +	0.08	2.76	0.031*	0.09
Color variety × Numerosity	8	232	1.22	0.287	0.04	0.23	0.985	0.01
Spatial arrangement × Color variety × Numerosity	8	232	2.73	0.007**	0.09	4.00	< 0.001***	0.12

+ *p* < 0.10, * *p* < 0.05, ** *p* < 0.01, *** *p* < 0.001

#### Response times (RTs)

A significant three-way interaction was observed (*F*(8, 232) = 2.73, *p* = 0.007, ${\upeta }_{\text{p}}^{2}$ = 0.09), suggesting that color variety's effect on RTs varied with spatial arrangement and numerosity. Simple effects analyses were conducted to explore the relationship between color variety and numerosity at each spatial arrangement level. For the clustered arrangement, we found significant main effects of both color variety (*F*(2, 58) = 5.60, *p* = 0.006, ${\upeta }_{\text{p}}^{2}$ = 0.16) and numerosity (*F*(4, 116) = 369.08, *p* < 0.001, ${\upeta }_{\text{p}}^{2}$ = 0.93), along with a significant color variety × numerosity interaction (*F*(8, 232) = 2.95, *p* = 0.004, ${\upeta }_{\text{p}}^{2}$ = 0.09). Post hoc analyses showed that color variety influenced RTs significantly for numerosity 41 (*F*(2, 58) = 7.15, *p* = 0.002, ${\upeta }_{\text{p}}^{2}$ = 0.20), with high- and medium-variety trials yielding shorter RTs than single-color trials by 7.05% (*t*(29) = 3.42, *p* = 0.006) and 3.32% (*t*(29) = 2.06, *p* = 0.049), respectively. High-variety trials were marginally faster than medium-variety (*t*(29) = 1.94, *p* = 0.062). For numerosity 13, the effect of color variety was marginally significant (*F*(2, 58) = 2.92, *p* = 0.062, ${\upeta }_{\text{p}}^{2}$ = 0.09), but follow-up tests showed no significant differences between variety conditions (all *p*s > 0.13). Other numerosities showed no effect of color variety (all *p*s > 0.12). For the random arrangement, significant main effects of color variety (*F*(2, 58) = 6.94, *p* = 0.002, ${\upeta }_{\text{p}}^{2}$ = 0.19) and numerosity(*F*(4, 116) = 335.63, *p* < 0.001, ${\upeta }_{\text{p}}^{2}$ = 0.92) were observed, but no interaction (*p* > 0.29). High- and medium-variety trials had longer RTs than single-color trials, with increases ranging from 1.47% to 5.79% for high-variety (*t*(29) = 3.97, *p* = 0.001) and from 1.18% to 4.85% for medium-variety (*t*(29) = 2.09, *p* = 0.046) trials. Additionally, RTs increased with numerosity across all conditions (all *p*s < 0.001), reflecting the demands of serial processing.

#### Error rates

A significant three-way interaction was observed (*F*(8, 232) = 4.00, *p* < 0.001, ${\upeta }_{\text{p}}^{2}$ = 0.12). We subsequently examined the simple interactions between color variety and numerosity for each spatial arrangement. For the clustered arrangement, significant main effects were observed for color variety (*F*(2, 58) = 7.34, *p* = 0.001, ${\upeta }_{\text{p}}^{2}$ = 0.20) and numerosity (*F*(4, 116) = 39.55, *p* < 0.001, ${\upeta }_{\text{p}}^{2}$ = 0.58), alongside a significant interaction (*F*(8, 232) = 2.53, *p* = 0.012, ${\upeta }_{\text{p}}^{2}$ = 0.08). Post hoc analyses revealed that color variety significantly influenced error rates for numerosity 41 (*F*(2, 58) = 8.66, *p* < 0.001, ${\upeta }_{\text{p}}^{2}$ = 0.23). High- and medium-variety trials had lower error rates than single-color trials (high-variety vs. single-color: *t*(29) = 3.11, *p* = 0.004; medium-variety vs. single-color: *t*(29) = 4.09, *p* < 0.001), corresponding to a 15.28% and 14.03% increase in accuracy, respectively. For numerosity 34, color variety's effect approached significance (*F*(2, 58) = 2.70, *p* = 0.075, ${\upeta }_{\text{p}}^{2}$ = 0.09), with high-variety trials reducing error rates compared to single-color trials (*t*(29) = 2.59, *p* = 0.045), and medium-variety trials showing a marginal reduction (*t*(29) = 1.71, *p* = 0.098). No significant effects were found for the other numerosities (all *p*s > 0.30). For the random arrangement, a significant main effect of numerosity was observed (*F*(4, 116) = 38.51, *p* < 0.001, ${\upeta }_{\text{p}}^{2}$ = 0.57), with error rates increasing as numerosity increased (all *p*s < 0.002). There was no significant main effect of color variety (*p* > 0.16) or interaction (*p* > 0.13).

### Discussion

The significant three-way interactions in both RT and error rate analyses highlight the complex effects of color on counting. In the clustered arrangement, multiple colors improved counting speed and accuracy, particularly for large numerosities. Conversely, in the random arrangement, multiple colors slowed down counting without affecting accuracy. Previous research indicates that display segmentation using spatial dividers enhances counting by facilitating focused attention (Li et al., 2018). In our study, color clusters acted as spatial dividers, segmenting displays into color-based areas and improving counting performance. These findings suggest that display segmentation – whether via spatial dividers or color clusters – optimizes the allocation of focused attention. However, in the random arrangement, multiple colors increased counting time, suggesting that color variation may disrupt focused attention by introducing redundancy and noise.

## General discussion

Estimation and counting represent distinct components of numerical cognition, relying on distributed and focused attention, respectively (Chong & Evans, 2011). This study examined how color influences both processes. We found that single-color arrays were perceived as less numerous than high-variety arrays, regardless of spatial arrangement. In contrast, counting performance improved with clustered colors but was impaired by random color distribution. These findings highlight that color impacts numerical processing, with spatial arrangement influencing serial counting but not parallel estimation.

In a previous study, Cordes et al. (2014) found that participants perceived more items in two-color displays than in single-color displays. They attributed this bias to operational momentum, suggesting that participants estimated each color subset separately and then summed these estimates. In our study, high-variety displays were similarly perceived as more numerous than single-color displays. However, this bias is unlikely due to operational momentum for the following reasons. First, summing estimates seems impractical with four and eight colors, as participants typically estimate only about three subsets simultaneously (Halberda et al., 2006). Second, Cordes et al.’s displays had predictable quantitative relationships between subsets (either 1:1 or 3:1), allowing participants to estimate one subset and extrapolate the total. In contrast, we used randomly determined quantities for each color, rendering such strategies ineffective. Thus, the observed bias in this study more likely arises from estimating the displays as a whole.

The perception of high-variety displays as significantly more numerous than single-color displays, and marginally more than medium-variety displays, may relate to the limits of visual attention (Pylyshyn & Storm, 1988) and visual working memory (Luck & Vogel, 1997), typically constrained to around four items. The eight colors in high-variety displays likely overwhelmed these limitations, creating an impression of greater numerosity. This numerical bias was observed only in high-variety displays, consistent with research showing no effect of increasing color categories from one to six (Halberda et al., 2006). While determining the exact boundary of this numerical bias is beyond this study's scope, future research could explore this boundary and potential individual differences.

We observed similar estimation performance in both clustered and random arrangements, indicating that the subjective perception of diversity is more closely tied to the overall richness of features than to their spatial arrangement during the early stages of visual processing. Moreover, participants appear to use diversity as a signal for the magnitude of quantity. This aligns with previous research showing that observers often associate greater quantity with richer diversity (Broniarczyk et al., 1998), and further extends this understanding by demonstrating that richer diversity also serves as a cue for greater numerosity. Additionally, the numerical bias elicited by color variety may be intrinsically linked to spillover bias, a phenomenon in which perceptions of diversity in a specific dimension can be influenced by the overall perceived diversity within a collection of stimuli (Mijalli et al., 2023). Our findings suggest the possibility that overall perceived diversity may function as a more generalized cue, influencing not only assessments of diversity along specific dimensions but also quantity judgments.

Our findings diverge from previous research suggesting that increased color entropy reduces perceived numerosity (Qu et al., 2022). Qu et al. (2022) proposed that higher entropy slows numerical information processing, leading to underestimation. Beyond differences in experimental stimuli – such as size, interstimulus distance, and the range of numerosity – a key distinction is that Qu et al. presented two arrays simultaneously, asking participants to determine which was more numerous, while our participants estimated the numerosity of a single array. There is evidence suggesting that reduced attention to stimuli increases the degree of underestimation (Kelley et al., 2017). In simultaneous arrays, attention may have been unevenly distributed, causing the more attention-grabbing array to be perceived as more numerous. Additionally, our task required participants to determine a specific value, likely introducing greater decision uncertainty compared to the binary comparisons used by Qu et al., leading our participants to rely more on color variety as a heuristic.

Another question to consider is whether participants based their estimates on density as a precursor to numerosity, calculating it as density multiplied by area (Dakin et al., 2011). If density played a role, our findings could suggest that color variety influences density perception. However, research indicates that density and numerosity perception are distinct mechanisms. Specifically, at low densities (below 0.25 items/deg^2^), numerosity estimation arises from direct perception of quantities, while at higher densities, it relies on the density-area relationship (Anobile et al., 2014). In our study, the numerosity range of 13–41 corresponds to densities between 0.08 and 0.26 items/deg^2^. Although the density for 41 circles slightly exceeds 0.25 items/deg^2^, most conditions featured lower densities, suggesting that participants likely engaged in direct numerosity perception. Given that luminance influences numerosity perception without affecting density (Ross & Burr, 2010) and that linking neighboring points in random-dot displays reduces perceived numbers without altering density judgments (He et al., 2009), further investigation is needed to determine whether our findings apply to higher density conditions.

Regarding the counting experiment, we found that spatial arrangement modulated the effect of color variety, resulting in distinct outcomes for clustered and random arrangements. Research on visual search indicates that increased diversity of distractors often impairs search performance, possibly due to increased noise with greater variability (Palmer et al., 2000). However, heterogeneity can enhance search performance when it provides useful information (Carter, 1982; Christ, 1975; Farmer & Taylor, 1980). Our findings align with this literature. Multicolor displays reduced counting speed with random color distribution, likely due to increased noise. In contrast, clustered colors improved counting efficiency, possibly by providing beneficial cues that facilitate focused attention allocation. This convergence with previous visual search studies suggests that counting and serial search share many processing similarities.

One might expect that randomly presented colors could aid counting by enhancing item individuation, a crucial process for distinguishing counted items from those not yet counted (Trick & Pylyshyn, 1993). However, in our experiment, each position corresponded to a single object, while each color represented multiple objects. This one-to-one mapping of position to object likely made spatial individuation more effective than color-based individuation, resulting in no advantage from increased color variety. Our findings suggest that when spatial coding is explicit, participants rely more on spatial information for individuation. Future research could explore whether random color arrangements facilitate counting in ambiguous spatial coding situations, such as closely packed or overlapping items.

In summary, this study provides compelling evidence that increasing color variety affects numerosity processing. Color variety influenced numerosity estimation independently of spatial arrangement, potentially engaging distributed attention for a global scan of variety while filtering out spatial details during parallel processing. In contrast, the effect of color variety on counting relies on spatial arrangement, indicating that color interacts with spatial layout to guide focused attention in serial processing. These findings underscore the necessity for theories of numerical cognition to account for feature variety and contextual factors, including spatial arrangement. Moreover, since color diversity is commonly encountered in everyday environments, our results may inform strategies to enhance behavior and improve adaptation to diverse contexts.

## Supplementary Information

Below is the link to the electronic supplementary material.

Supplementary file1 (DOCX 17 KB)

## Data Availability

The data and materials are available on the Open Science Framework and can be accessed at https://osf.io/hrgd3/?view_only=2ea69a11293244849e4e9dff565abc70

## Code availability

The analysis code is also available on the Open Science Framework and can be accessed at https://osf.io/hrgd3/?view_only=2ea69a11293244849e4e9dff565abc70

## Declarations

### Open practices statement

The data and materials for both experiments are available via the Open Science Framework at https://osf.io/hrgd3/?view_only=2ea69a11293244849e4e9dff565abc70. Neither experiment was preregistered.
