# Supplementary material for: The influence of increasing color variety on numerosity estimation and counting
Source: Psychon Bull Rev. 2025 Jan 3;32(3):1391–401. doi: 10.3758/s13423-024-02625-x (PMC12092485; doi:10.3758/s13423-024-02625-x)
Supplement: Supplementary file 1 — Supplementary file1 (DOCX 17 KB) [file 13423_2024_2625_MOESM1_ESM.docx]

Appendix

Table A1 shows the mean standard deviations of estimation errors across participants, reflecting the consistency and precision of the estimations. A three-way repeated measures ANOVA with spatial arrangement (clustered, random), color variety (single, medium, high variety), and numerosity (13, 20, 27, 34, 41) as within-participant factors yielded a significant main effect of numerosity (*F*(4, 116) = 37.14, *p* < .001, $\eta_{p}^{2}$ = .56). Post hoc contrasts showed that variability for numerosity 13 was significantly lower than for all other numerosities (all *p*s < .001). Numerosity 20 also showed lower variability than 27, 34, and 41(all *p*s < .022), while no significant differences were found among numerosities 27, 34, and 41. These results suggest that smaller numerosities are associated with reduced variability in estimation errors. However, no significant effects of color variety (*p* > .57) or interactions (all *p*s > .65) were found, indicating similar estimation consistency and precision across color variety conditions.

Table A1 Mean standard deviations of estimation errors. Standard errors are shown in parentheses.

|  | 13 | 20 | 27 | 34 | 40 |
| --- | --- | --- | --- | --- | --- |
| Clustered |  |  |  |  |  |
| Single color | 3.21 (0.23) | 4.05 (0.22) | 4.71 (0.24) | 4.92 (0.31) | 5.00 (0.33) |
| Medium variety | 3.01 (0.22) | 4.05 (0.23) | 4.56 (0.23) | 5.00 (0.21) | 5.04 (0.36) |
| High variety | 3.00 (0.23) | 4.21 (0.29) | 4.77 (0.30) | 5.17 (0.34) | 4.89 (0.26) |
| Random |  |  |  |  |  |
| Single color | 3.03 (0.22) | 4.07 (0.20) | 4.62 (0.31) | 5.04 (0.25) | 4.59 (0.30) |
| Medium variety | 3.17 (0.23) | 4.35 (0.27) | 4.52 (0.25) | 5.00 (0.25) | 4.79 (0.28) |
| High variety | 3.01 (0.22) | 4.27 (0.22) | 4.94 (0.38) | 4.91 (0.32) | 4.86 (0.35) |
